# Alpha Tocopherol-Induced Modulations in the Morphophysiological Attributes of Okra Under Saline Conditions

**DOI:** 10.3389/fpls.2021.800251

**Published:** 2021-12-21

**Authors:** Maria Naqve, Xiukang Wang, Muhammad Shahbaz, Athar Mahmood, Safura Bibi, Sajid Fiaz

**Affiliations:** ^1^Department of Botany, University of Agriculture, Faisalabad, Pakistan; ^2^College of Life Sciences, Yan’an University, Yan’an, China; ^3^Department of Agronomy, University of Agriculture, Faisalabad, Pakistan; ^4^Department of Plant Breeding and Genetics, The University of Haripur, Haripur, Pakistan

**Keywords:** alpha tocopherol, salinity, okra, biomass, photosynthetic attributes

## Abstract

Foliar spray of antioxidants is a pragmatic approach to combat various effects of salinity stress in agricultural crops. A pot trial was conducted to examine the effect of exogenously applied α-tocopherol (α-Toc) as foliar spray to induce morpho-physiological modulations in two varieties (Noori and Sabzpari) of okra grown under salt stress conditions (0 mM and 100 mM NaCl). After 36 days of salinity treatments, four levels (0, 100, 200 and 300 mg L^–1^) of α-tocopherol were sprayed. Salt stress significantly reduced root and shoot fresh and dry biomass, photosynthesis rate (*A*), transpiration rate (*E*), water use efficiency (*A*/*E*), stomatal conductance, internal CO_2_ concentration (*C*_*i*_)and *C*_*i*_/*C_*a*_)*, and photosynthetic pigments. Foliar spray of α-tocopherol proved effective in improving the growth of okra by significantly enhancing root dry weight, root length, shoot fresh weight, shoot length, Chl. *a*, Chl. *b*, Total chl., β-Car., Total Car., *A, E, A*/*E, C_*i*,_* and *C*_*i*_/*C*_*a*_, leaf and root Ca^2+^ and K^+^ ion content, total soluble sugars, non-reducing sugars and total soluble protein content by significantly reducing root Na^+^ ion content. The Okra variety Noori performed better than Sabzpari in the examined attributes, and 300 mg L^–1^ application of α-tocopherol was more pronounced in improving the growth of okra by alleviating salinity effects. Therefore, the use of α-tocopherol (300 mg L^–1^) as a foliar spray is recommended to improve okra production in saline soils.

## Graphical Abstract

Salinity stress mitigation by the application of alpha tocopherol in okra.

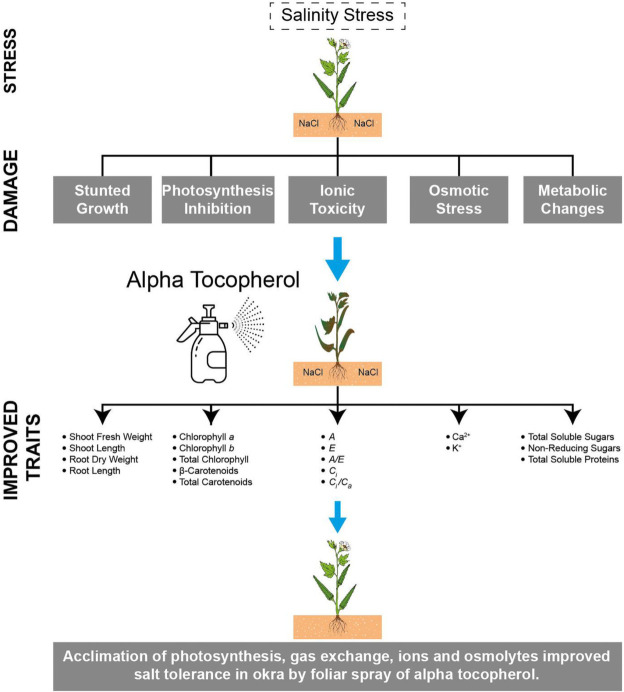

## Introduction

Okra [*Abelmoschus esculentus* (L.) Moench] is the most popular mallow crop, and its capsules and leaves are mainly consumed as vegetables, capsules are rich source of vitamin A, B6, C and K (Aboyeji et al., 2021). potassium, calcium, carbohydrates, unsaturated fatty acids such as linolenic acid, oleic acid and minerals along with enormous amounts of mucilage with medicinal properties (Naqve et al., 2018; Schafleitner et al., 2021). Salt stress significantly reduced K^+^ and Ca^2+^ ions and yield and increased H_2_O_2_, malondialdehyde (MDA), Na^+^, glycine betaine (GB), total free proline, total phenolics, and the activities of catalase (CAT), guaiacol peroxidase (GPX), and protease in Noori (salt tolerant) and Sabzpari (salt sensitive) (Naqve et al., 2018).

Okra is classified as a moderately sensitive crop to salinity stress by the Food and Agriculture Organization (FAO), and it is susceptible to salinity stress in areas where summers are dry (Elshaikh et al., 2018). Based on germination, growth and yield characteristics, salinity tolerant (8 dSm^–1^) varieties of okra (OH-713, OH-139, OH-138, OH-2324 and OH-001) has been reported whereas the sensitive genotypes include Kiran, Okra-1548, Ikra-3, Sabzpari, Okra-7100, Sitara-9101, and Okra-7080 (sensitive 4 dSm^–1^). However, increasing salinity stress decreases the per hectare productivity of okra (Mushtaq et al., 2020). Among abiotic stresses, salinity is a major threat to crop productivity (Ahmad et al., 2019). Cultivated land in Asia is expected to be affected by 50% by soil salinization by the year 2050 (Khan et al., 2019). Soil salinity (2 to 3 dSm^–1^) aggravates the fertility of soil and drastically decreases crop yield (Shilev, 2020). Among all other soluble salts causing adverse effects on plants, NaCl is most dominant, and a high concentration of NaCl hampers various plant processes, including seed germination, root growth, nutrient uptake, and photosynthesis (Naveed et al., 2020). Reduced root and shoot growth are primary symptoms of salinity effects (Liu et al., 2020). Salinity affects plant growth and yield by reducing the photosynthesis rate, biomass, and water use efficiency (Moles et al., 2019). Increased levels of saline stress decrease stomatal conductance, photosynthesis, and chlorophyll content in the leaves of plants (Sun et al., 2018).

Due to reduced plant height and the number of leaves and root and shoot biomass under saline conditions, plant growth is severely affected (Naqve and Shahbaz, 2019). Reduced uptake of CO_2_ is associated with increased salinity, which alters stomatal functions, photosynthesis, and transpiration (Hussain et al., 2018). Salt stress alters the ionic status of plants, which aggravates enzymatic activities, cellular turgor, and protein biogenesis by shunting osmotic stress (Chokshi et al., 2017). Oxidative stress is also a major threat to crop production under saline regimes due to the overproduction of reactive oxygen species (ROS) (Mehla et al., 2017). To resist salinity, plants adapt various responses at the molecular level by the upregulation of plant hormones, organic osmotica and antioxidants (Soni et al., 2017). Primarily, plants cope with salinity-induced oxidative bursts by up-regulating defensive antioxidants, including catalase, peroxidases, ascorbic acid, flavonoids, phenolics and α-tocopherol (Hasanuzzaman et al., 2020). Maintaining optimal ROS concentrations by ROS quenchers regulates various processes essential for the growth and development of plants (Mittler, 2017). Thus, exogenous application of these compounds can be helpful for plants to resist salinity, which can be a potentially simple, economical, and culturally feasible approach compared with introgression and genetic engineering (Sadiq et al., 2019). Foliar spraying of α-tocopherol is one such approach to improve plant growth under salinity stress (Surówka et al., 2020). Tocopherols are members of the vitamin E family and consist of alpha, beta, gamma, and delta forms (Shahidi and De Camargo, 2016). Alpha tocopherol is more active than all other categories of vitamin E, as it protects photosystem II and lipid membranes in chloroplasts from salinity-induced damage (Sadiq et al., 2019; Surówka et al., 2020). α-tocopherol also protects chloroplasts by quenching ROS, thus improving photosynthetic efficiency in plants (Hasanuzzaman et al., 2020). Moreover, it also protects plants by quenching free radical species produced under salinity stress by enhancing the production of other antioxidants (Semida et al., 2014), thus improving the growth and yield of plants (Surówka et al., 2020)The agricultural economy of Pakistan is important for the food of 207 million people, which is threatened by salinity. The total area of Pakistan is 79.6 mha, of which 22 mha is cultivated land and 6.28 mha is affected by salt (GOP, 2020). Salinity has serious socioeconomic issues in the agricultural communities of the country. Selection and breeding for improved tolerance against salinity in different vegetables and crops is considered a high priority due to economic benefits and is the best alternative for the farming community. Okra is a commonly consumed vegetable in Pakistan, but its per ha productivity is being reduced due to the increasing land salinity problem in Pakistan. Hence, it is urgent to introduce cost-effective strategies for farmers, such as foliar spraying of compounds that are naturally produced by plants such as α-tocopherol, to combat salinity problems. These strategies can be more effective than the application of synthetic fertilizers that are organic in nature. Such compounds as α-tocopherol are beneficial for plants and humans as well. Therefore, the present study aimed to investigate the effect of foliar spray application of α-Toc on the morpho-physiological attributes of okra under salt stress.

## Materials and Methods

To evaluate whether foliar spray of α-tocopherol could alleviate salinity-induced negative effects on okra, the current experiment was laid out in the research area of the Old Botanical Garden, University of Agriculture Faisalabad, Pakistan. Okra varieties (Sabzpari and Noori) differing in salinity tolerance were selected, and seeds of selected varieties were obtained from the Ayub Agricultural Research Institute (AARI), Faisalabad, Pakistan.

The experiment was conducted twice in plastic pots (24 cm wide and 30 cm deep), each containing 10 kg well-washed dry sand. In each pot, six plants were maintained after thinning, and the experiment was carried out with four replicates under a completely randomized design (CRD). After sowing full strength (2 L per pot), Hoagland’s nutrient solution was supplied weekly. During the experiment, salinity stress was maintained by NaCl levels (0 mM and 100 mM) in full-strength Hoagland’s nutrient solution after 21 days of germination. Concentration of NaCl was maintained in aliquot parts of 50 mM to prevent osmotic shock. Thus, salinity level was maintained in two phases. In first phase 50 mM NaCl level was maintained and after 2 days of this level, the required level of 100 mM was maintained. Foliar fertigation of α-tocopherol concentrations [0 (distilled water), 100, 200 and 300 mg L^–1^] was performed on 36-day-old plants. Each pot was supplied with a 25 mL solution of each concentration of α-tocopherol just before sunset for maximum absorption and to avoid sunburn of leaves. For enhancement at absorption, Tween 20 (0.1%) was used as the surfactant. After 3 weeks of foliar spray from each replicate, two plants were uprooted carefully and washed, and data were recorded.

### Photosynthetic Pigments

The protocol described by Arnon (1949) was followed to measure the concentrations of various photosynthetic pigments. Fresh leaves (0.5 g) were extracted in 80% acetone (10 ml). Chlorophyll was extracted by thoroughly grinding it in a mortar pesto. The absorbance of photosynthetic pigments was read at OD 663, OD 645, OD 505, OD 453, and OD 470 nm by using a spectrophotometer (IRMECO-U2020) and, calculations were made by using following formulae


Chl.a(mgml)-1=[12.7(OD663)-2.69(OD645)]×[V/(1000×W)]

Chl.b(mgml)-1=[22.9(OD645)-4.68(OD663)]×[V/(1000×W)]

Carotenoids(gml)-1=[A/carEm100%]×100

A=car[(OD480)+0.114(OD663)-0.638(OD645)]

Where,

V is the volume of the sample.

W is the weight of fresh tissue taken for extraction.

Em100% = 2500.

### Growth Attributes

After calculating fresh masses of roots and shoots, the same plant was oven-dried using an electric oven at 70°C for 48 h, and their dry masses were calculated using a digital electronic balance.

### Leaf Gas Exchange Characteristics

For the assessment of leaf gaseous exchange traits, an LCA-4 portable infrared gas analyzer (IRGA) was used with specifications of ambient pressure (P), 98.5 kPa, gas flow rate, (U) 252 μmol S^–1^, ambient CO_2_ concentration 350 μmol mol^–1^, water vapor pressure, 6.0–9.0 mbar, temperature, 28–32°C, relative humidity, 41.1% and air flow/unit leaf area (Us) 22.05 mol m^–2^ S^–1^. Data were recorded during 9: 00 A.M. to 12: 00 P.M. under suitable weather conditions.

### Nutrients Analysis for Leaf and Root

Dried leaf and root tissues were used separately for the analysis of nutrients (Na, K and Ca) by following the acid digestion protocol as proposed by Wolf (1982).

### Acid Digestion Method

Leaf- and root-dried material (0.1 g) was digested in 2.5 ml of conc. H_2_SO_4_ overnight at room temperature in digestion flasks. To it was then added one mL of 35% H_2_O_2_ and the flask was heated at 350°C until the production of fumes. The heating process continued for approximately 30 min, then 1 mL of 35% H_2_O_2_ was again added while the heating process continued. This process was repeated until the mixture became colorless, and it was then diluted with distilled water up to 50 mL and filtered. The filtrate was used for the determination of mineral ions by using a flame photometer.

### Total Soluble Proteins

Fresh plant leaves (0.5 g) were crushed with 10 ml (50 mM) potassium phosphate buffer with pH 7.0 in a chilled environment, and the extract (5 μL) was then homogenized with 1 ml of Bradford dye and 0.1 N HCl. The optical density was read at 595 nm by following the protocol of Bradford (1976).

### Sugars Content

The procedure of Dubois et al. (1951) was followed to estimate the total soluble sugar content. The reaction mixture was prepared by adding (0.5mL) sample, 5% phenol solution (0.25 mL) and 96% sulfuric acid (1.25 mL), and the absorbance was measured at 490 nm by using an IRMECO-U2020 spectrophotometer.

For the determination of reducing sugars, reaction solution was set in test tubes by adding sample, H_2_O and DNS reagent. Absorbance was noted at 540 nm using an IRMECO-U2020 spectrophotometer by following (Miller et al., 1973). In this way, the non-reducing sugars were determined by the difference in reducing and total soluble sugars.

### Statistical Analysis

Collected data were investigated for analysis of variance of data for all the recorded traits by following Snedecor and Cochran (1980), and the means were compared using least significant difference (LSD) at a probability level of 5% by using COSTAT software.

## Results

### Growth Attributes

**FIGURE 1:**
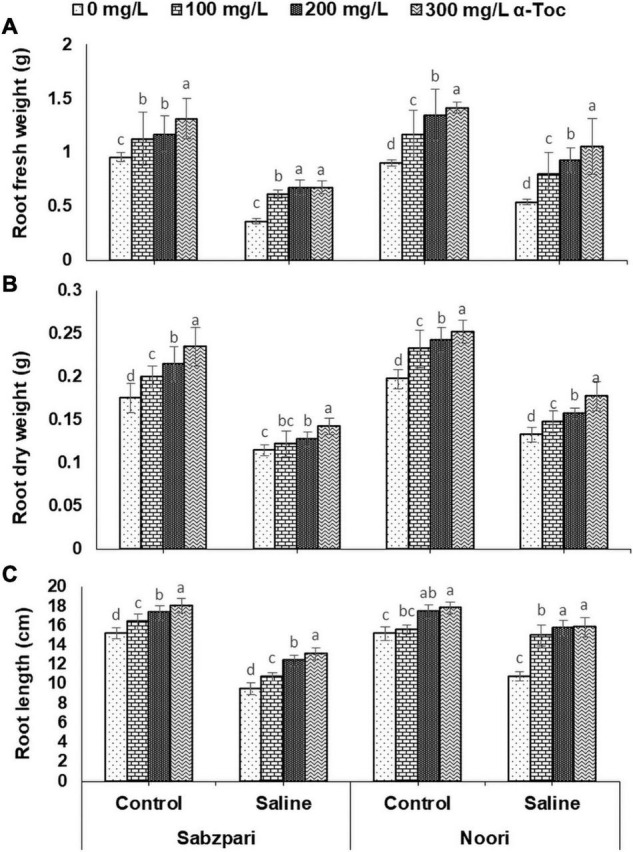
Root fresh weight **(A)**, Root dry weight **(B)**, Root length **(C)** of okra varieties sprayed with different levels of α-tocopherol under saline conditions. Values represent means ± S.D. Significant differences among row spacing were measured by the least significant difference (LSD) at *p* > 0.05 and indicated by different letters.

The root fresh weight (RFW) of okra varieties was significantly decreased by salinity stress in the present study. The data showed that Noori exhibited considerably higher RFW than Sabzpari. Foliar spray of α-tocopherol did not significantly affect this attribute under the control and stress treatments (Table 1 and Figure 1A).

**TABLE 1:** Analysis of variance (mean squares) for growth traits and photosynthetic attributes of okra plants treated with α-tocopherol as foliar spray under saline and non-saline conditions.

Source	df	RFW	RDW	RL	SFW	SDW	SL	Chl. *A*	Chl. *B*	Chl. *a*/*b*	Total Chl.	β-Car.	Total Car.
V	1	0.81*	0.01***	4.05ns	20.47**	0.66**	503.44***	15131.8***	360.63***	0.001ns	21780.12***	2.45*	0.86ns
S	1	4.0***	0.10***	103.78***	152.76***	1.17***	1018.40***	19117.6***	1633.23***	34.252***	29555.42***	2.79*	252.55***
α-Toc	3	0.25ns	0.003*	36.83***	16.43***	0.17 ns	72.54**	9276.5***	225.12***	0.404ns	5308.77***	2.37**	16.10**
V x S	1	0.02ns	0.001ns	6.06ns	1.17ns	0.03 ns	2.28ns	289.1ns	0.18 ns	0.019ns	5305***	1.01ns	8.55ns
V x α-Toc	3	0.03ns	2.72 ns	0.33ns	0.67ns	0.02 ns	8.98ns	114.5ns	22.53**	2.096ns	769.03*	1.69*	14.65**
S x α-Toc	3	0.02ns	5.72 ns	2.35ns	1.51ns	0.02 ns	13.88ns	326.9ns	3.32ns	3.123*	194.70ns	1.50*	4.88ns
V x S x α-Toc	3	0.04ns	5.66 ns	1.39ns	1.84ns	0.01 ns	7.01ns	1063.7*	37.95***	3.563*	661.16*	1.33ns	12.20*
Error	48	0.13	8.02	4.05ns	1.84	0.08	15.19	292.50	5.23	1.050	264.06	422.67	0.002
V (LSD 0.05%)		0.18	0.014	1.00	0.68	0.14	1.95	8.59	1.15	0.51	6.90	0.36	0.89
S (LSD 0.05%)		0.18	0.014	1.00	0.68	0.14	1.95	8.59	1.15	0.51	6.90	0.36	0.89
α-Toc (LSD 0.05%)		0.25	0.02	1.42	0.96	0.20	2.77	12.15	1.62	0.72	9.76	0.5	1.26

*, **, and ***Significant at 0.05, 0.01 and 0.001 levels, respectively; ns, non-significant; V, Varieties; S, Salinity; α-toc, Alpha tocopherol; RFW, Root Fresh Weight; RDW, Root Dry Weight; SFW, Shoot Fresh Weight; SDW, Shoot Dry Weight; SL, Shoot Length; Chl. a, Chlorophyll a; Chl.b, Chlorophyll b; Chl. a/b, Chlorophyll a/b; Total Chl., Total Chlorophyll; β-Car., Beta Carotenoids; Total Car., Total Carotenoids.

The current study revealed that salt stress induced a significant reduction in the RDW of both okra varieties. Noori was superior in RDW than that of Sabzpari. Sprays of α-tocopherol markedly enhanced the RDW of salt-stressed and non-stressed plants. Furthermore, a higher concentration (300 mg L^–1^) of α-tocopherol increased the RDW of both varieties (Table 1 and Figure 1B).

A significant reduction was observed in the root length (RL) of both tested varieties of okra. However, Noori produced more RL than Sabzpari. Foliar supplementation with α-tocopherol significantly improved the RL of okra. Additionally, foliage spray of α-tocopherol (200 mg L^–1^ and 300 mg L^–1^) improved RL under salinity. Overall, a 300 mg L^–1^ concentration of α-tocopherol showed a pronounced effect in increasing the RL of okra varieties under stressed and non-stressed conditions (Table 1 and Figure 1C).

**FIGURE 2:**
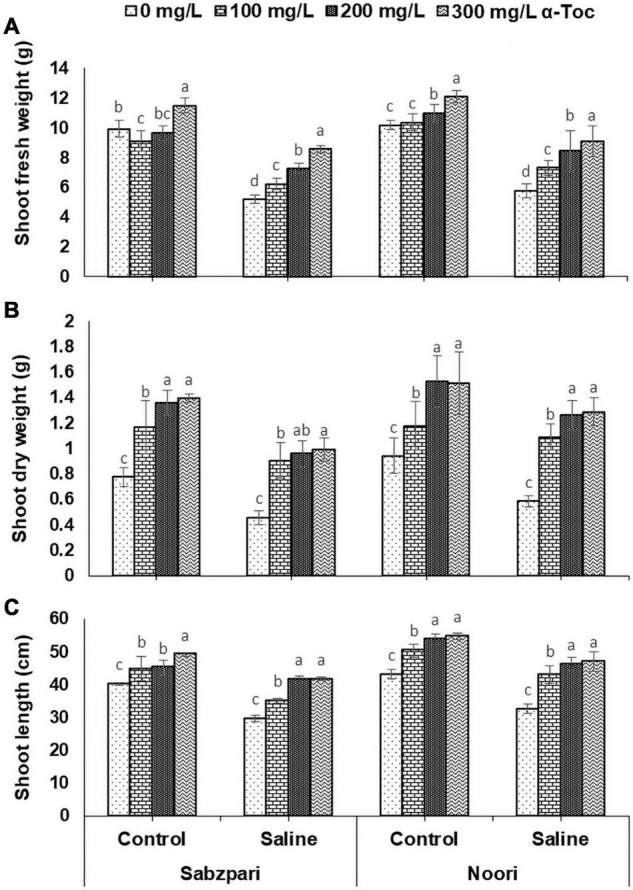
Shoot fresh weight **(A)**, Shoot dry weight **(B)** and Shoot length **(C)** of okra varieties sprayed with different levels of α-tocopherol under saline conditions. Values represent means ± S.D. Significant differences among row spacing were measured by the least significant difference (LSD) at *p* > 0.05 and indicated by different letters.

The data revealed that saline (NaCl) stress significantly reduced the shoot fresh weight (SFW) of the tested okra varieties. However, Noori was superior to Sabzpari with respect to SFW. Foliar fertigation of α-tocopherol significantly increased the SFW of both varieties. However, among the effects of various levels of α-tocopherol under salt stress conditions, 300 mg L^–1^ was more effective in enhancing shoot growth than the other levels (Table 1 and Figure 2A).

Salt stress significantly reduced the shoot dry weight of both tested varieties, and this reduction was more pronounced in Sabzpari than in Noori. Surprisingly, foliar application of α-tocopherol did not affect the shoot dry weight (SDW) of either tested variety under either salinity treatment. However, 200 and 300 mg L^–1^ levels of α-tocopherol spray showed little improvement in the SDW of stressed and non-stressed plants (Table 1 and Figure 2B).

A reduction in shoot length (SL) of both tested varieties was observed under salinity stress (100 mM NaCl). However, foliar spraying with higher levels of α-tocopherol (300 mg L^–1^) caused a significant increase in the SL of the tested varieties under stressed and non-stressed conditions. This increase in SL was more pronounced in Noori than in Sabzpari (Table 1 and Figure 2C).

### Photosynthetic Pigments

**FIGURE 3:**
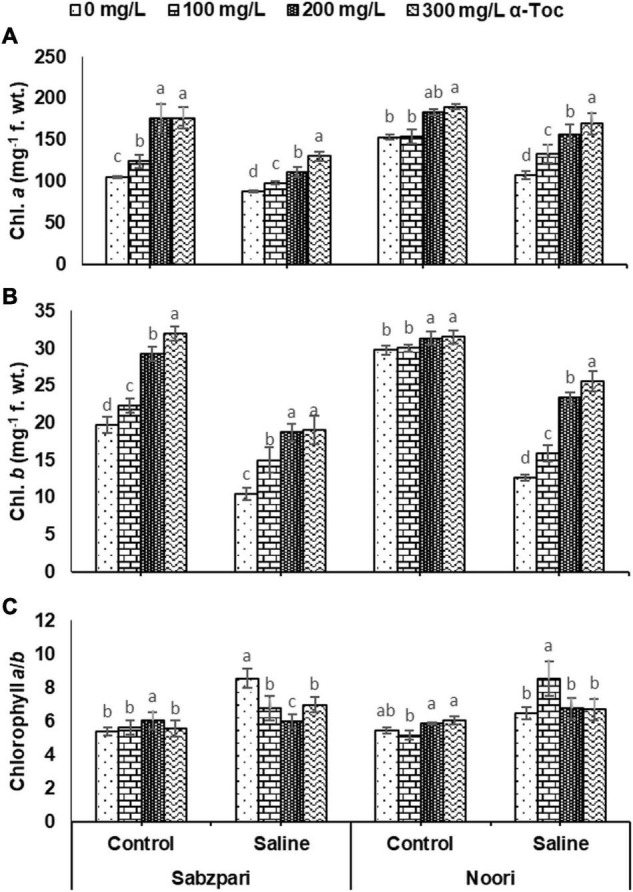
Chl. *a* **(A),** Chl. *b* **(B)**, Chl. *a*/*b* **(C)** of okra varieties sprayed with different levels of α-tocopherol under saline conditions. Values represent means ± S.D. Significant differences among row spacing were measured by the least significant difference (LSD) at *p* > 0.05 and indicated by different letters.

**FIGURE 4:**
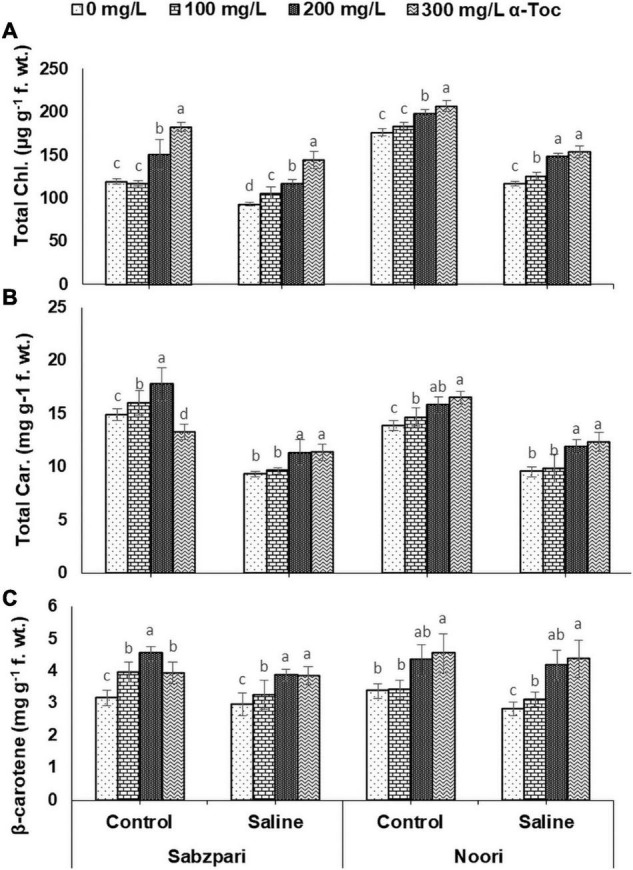
Total Chl. **(A)**, Total Car. **(B)**, β- Carotene **(C)** of okra varieties sprayed with different levels of α-tocopherol under saline conditions. Values represent means ± S.D. Significant differences among row spacing were measured by the least significant difference (LSD) at *p* > 0.05 and indicated by different letters.

Root medium salinity stress remarkably suppressed chlorophyll *a*, *b* and β-carotene contents. The response of both okra varieties was also significant in terms of these traits. Moreover, varying concentrations of α-tocopherol applied as foliar spray markedly boosted the level of these pigments in both varieties. While 300 mg L^–1^ improved pigment concentrations under stressed and non-stressed plants of both okra varieties, Noori showed relatively higher accumulation of these pigments than Sabzpari under saline and non-saline conditions. Significant interactions of NaCl levels, α-tocopherol application, and the tested varieties were observed in terms of chlorophyll *a* and *b* content. The Okra variety Noori reached higher concentrations of both pigments at a 300 mg L^–1^ spray of α-tocopherol under salinity, whereas the minimum concentration of these pigments was recorded in Sabzpari (Table 1 and Figures 3A,B, 4C).

A significant effect of salinity was observed on the chlorophyll *a*/*b* ratio (Chl. *a*/*b*). Both okra varieties showed comparable values of Chl. *a*/*b*. The data of the present study revealed that exogenously applied α-tocopherol did not show any change in this ratio. A significant interactive effect was recorded between NaCl stress and foliar application of α-tocopherol. At the 100 mg L^–1^ level of α-tocopherol, okra variety Noori performed better in terms of Chl. *a*/*b* (Table 1 and Figure 3C).

Total chlorophyll (Total Chl.) content was significantly decreased under salt (NaCl) stress. A significant impact of α-tocopherol (300 mg L^–1^) application was noticed regarding total chlorophyll under stressed and non-stressed conditions. A significant interactive effect of variety and α-tocopherol was noticed with respect to the total chlorophyll content. Noori showed the highest total chlorophyll content at a higher level of α-tocopherol (300 mg L^–1^) (Table 1 and Figure 4A).

In the current study, a significant reduction was observed in total carotenoids (Total Car.) content in both varieties under salt stress than in non-stressed plants. However, foliar application of varying levels of α-tocopherol enhanced the total carotenoid content of both varieties under saline- and salinity-free treatments. The variety Noori performed better with respect to total carotenoids content than Sabzpari at 300 mg L^–1^ foliar spray of α-tocopherol under 100 mM NaCl stress (Table 1 and Figure 4B).

### Gas Exchange Characteristics

**FIGURE 5:**
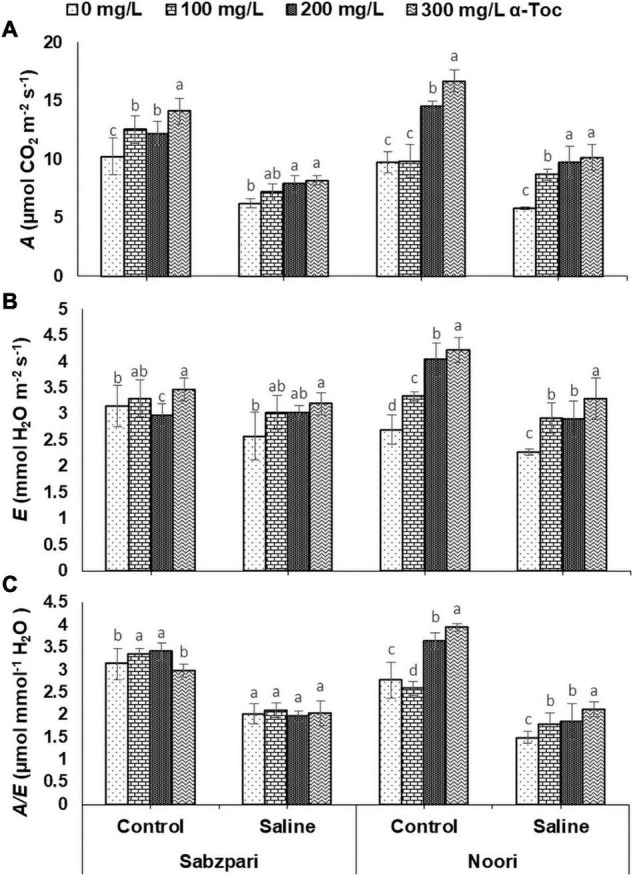
Photosynthetic rate *A* **(A)**, Transpiration rate *E* **(B)**, Water use efficiency *A*/*E* **(C)**, of okra varieties sprayed with different levels of α-tocopherol under saline conditions. Values represent means ± S.D. Significant differences among row spacing were measured by the least significant difference (LSD) at *p* > 0.05 and indicated by different letters.

Salt stress (100 mM NaCl) significantly reduced photosynthetic traits. However, significant improvement was observed in transpiration rate (*E*), photosynthesis rate (*A*) and water use efficiency (*A*/*E*) after foliar spray of α-tocopherol (300 mg L^–1^) under saline and controlled conditions. Noori was superior to Sabzpari in these attributes. A significant interaction was observed among the tested varieties and treatments regarding *A*, and the highest *A* level was determined in the variety Noori combined with 300 mg L^–1^ α-tocopherol under 100 mM NaCl (Table 2 and Figures 5A–C).

**TABLE 2:** Analysis of variance (mean squares) for leaf gas exchange attributes of okra plants treated with α-tocopherol as foliar spray under saline and non-saline conditions.

Source	df	*A*	*E*	*A*/*E*	*g*_*s*_	*C*_*i*_	*C*_*i*_/*C*_*a*_
V	1	5.02ns	0.23ns	0.16ns	976.56ns	1881.39*	0.025***
S	1	285.94***	3.97**	27.29***	27639.06 ***	9225.60***	0.002ns
α-Toc	3	16.61*	2.06**	0.64*	1926.56 ***	6984.22***	0.078***
V x S	1	1.65ns	0.85ns	0.22ns	976.56ns	6756.84***	0.024**
V x α-Toc	3	29.14**	0.64ns	0.97**	64.06ns	861.60ns	0.005ns
S x α-Toc	3	19.86*	0.05ns	0.26ns	68.22ns	6573.09***	0.188 ***
V x S x α-Toc	3	16.82*	0.34ns	0.30ns	305.72ns	603.31ns	0.024 ***
Error	48	4.73	0.34	0.21	264.06	422.67	0.002
V (LSD 0.05%)		1.09	0.29	0.23	8.16	10.33	0.02
S (LSD 0.05%)		1.09	0.29	0.23	8.16	10.33	0.02
α-Toc (LSD 0.05%)		1.54	0.41	0.32	11.55	14.61	0.03

*, **, and ***Significant at 0.05, 0.01, and 0.001 levels, respectively; ns, non-significant; V, Varieties; S, Salinity; α-toc, Alpha tocopherol: Photosynthetic rate (A), Transpiration rate (E), Water use efficiency (A/E), Stomatal conductance (g_s_), Internal CO_2_ concentration (C_i_), Ratio of internal and ambient CO_2_ concentration (C_i_/C_a_).

**FIGURE 6:**
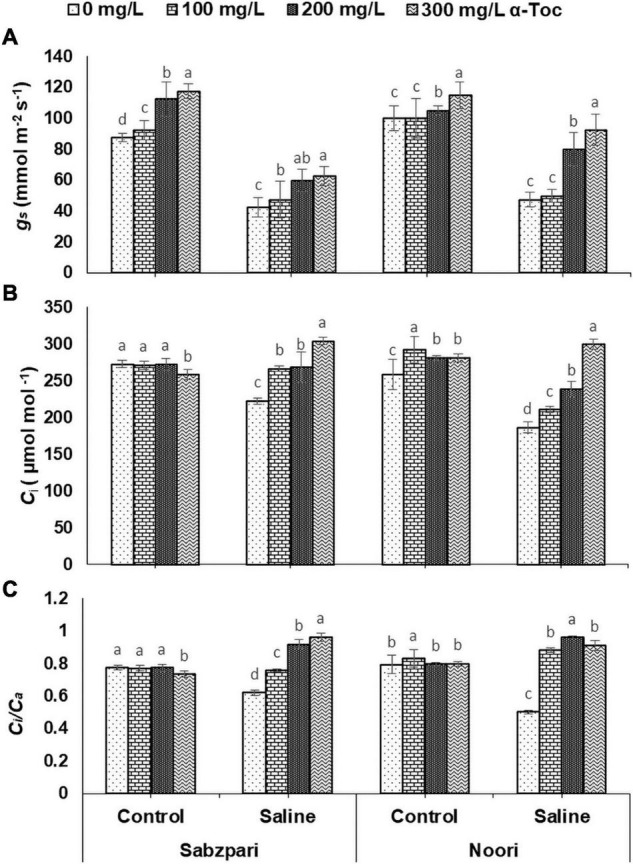
Stomatal conductance *g*_*s*_ **(A)**, Internal CO_2_ concentration *C*_*i*_ **(B)** *C*_*i*_/*C*_*a*_ **(C)** of okra varieties sprayed with different levels of α-tocopherol under saline conditions. Values represent means ± S.D. Significant differences among row spacing were measured by the least significant difference (LSD) at *p* > 0.05 and indicated by different letters.

A significant reduction was observed in the stomatal conductance (*g*_*s*_) of the tested okra plants under the saline regime. The Okra variety Noori showed higher stomatal conductance (*g*_*s*_) than Sabzpari under saline conditions. In contrast, a similar performance of both varieties was observed regarding stomatal conductance under non-stressed conditions. Exogenous application of α-tocopherol (300 mg L^–1^) as foliar spray had a remarkable effect on stomatal conductance (*g*_*s*_) in both varieties (Table 2 and Figure 6A).

The substomatal internal CO_2_ concentration (*C*_*i*_) significantly decreased in both okra varieties under salinity. Both tested varieties exhibited similar performance toward substomatal internal CO_2_ concentrations. Moreover, foliar application of α-tocopherol significantly improved the *C*_*i*_ of the tested varieties under salt stress. A higher level (300 mg L^–1^) of foliar α-tocopherol application clearly improved the *C*_*i*_ of both varieties under stressed conditions (Table 2 and Figure 6B).

Similarly, a significant effect on the ratio of internal and ambient CO_2_ *C*_*i*_/*C*_*a*_ of both varieties was also observed. Foliar fertigation of tested plants with α-tocopherol markedly improved the *C*_*i*_/*C*_*a*_ concentration in both okra varieties (Figure 6C). Overall, (200 and 300 mg L^–1^) spray of α-tocopherol proved better in enhancing *C*_*i*_/*C*_*a*_ in both varieties under saline regime. The interactive effects of the Noori variety with foliar α-tocopherol (200 mg L^–^1) spray under 100 mM NaCl stress exhibited a higher combined effect than the other treatments (Table 2 and Figure 6B).

### Ionic Content

**FIGURE 7:**
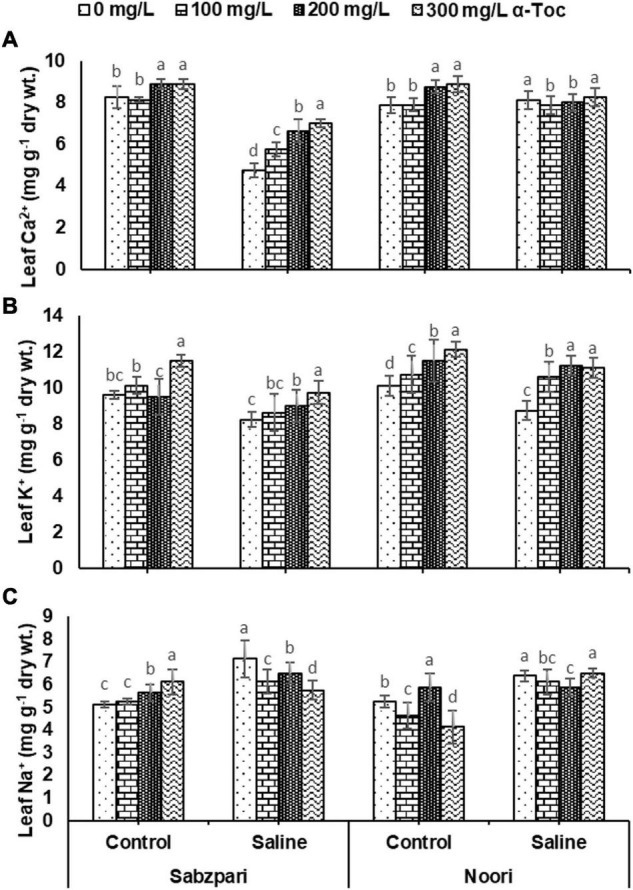
Leaf Ca^2+^ **(A)**, Leaf K^+^ **(B)** Leaf Na^+^ **(C)** of okra varieties sprayed with different levels of α-tocopherol under saline conditions Values represent means ± S.D. Significant differences among row spacing were measured by the least significant difference (LSD) at *p* > 0.05 and indicated by different letters.

The application of salt remarkably decreased leaf Ca^2+^ and K^+^ contents in both okra varieties. Tested okra varieties (Noori and Sabzpari) had no significant impact on leaf Ca^2+^ content, while in terms of leaf K^+^ content, both varieties differed significantly, as Noori accumulated more K^+^ than Sabzpari. The α-tocopherol spray significantly enhanced the leaf Ca^2+^ and K^+^ contents of both varieties of okra under saline and non-saline regimes (Table 3 and Figures 7A,B).

**TABLE 3:** Analysis of variance (mean squares) for leaf gas exchange attributes of okra plants treated with α-tocopherol as foliar spray under saline and non-saline conditions.

Source	df	Total soluble proteins	Reducing sugars	Non-reducing sugars	Total soluble sugars	Leaf Ca^2+^	Leaf K^+^	Leaf Na^+^	Root Ca^2+^	Root K^+^	Root Na^+^
V	1	28532.84***	22.65***	15.59ns	10.95ns	0.47ns	26.26**	3.28ns	1.56ns	0.66ns	4.25ns
S	1	8883.06***	5.51*	113.63***	61.02***	17.53***	19.14**	14.53 ***	1ns	98.75 ***	38.28 ***
α-Toc	3	607.43ns	2.53ns	129.17***	90.82***	4.48***	11.54**	0.58ns	1.38ns	2.72ns	10.50 ***
V x S	1	70.84ns	18.36***	121.27***	55.90**	13.59***	9.76ns	0.19ns	4.51 *	3.28ns	21.97 ***
V x α-Toc	3	48183.32***	0.30ns	20.74**	11.88ns	0.53ns	3.08 ns	0.23ns	0.82ns	1.04ns	11.47 ***
S x α-Toc	3	18282.91***	6.08**	94.19***	52.95***	2.82**	3.35 ns	0.50ns	1.01ns	3.03ns	8.62 **
V x S x α-Toc	3	7053.95***	0.50ns	38.03***	35.97***	1.13ns	1.02 ns	3.72 *	0.04ns	2.31ns	7.46 **
Error	48	404.83	1.13	4.44	4.55	0.62	2.42	0.91	1.05	2.00	1.56
V (LSD 0.05%)		10.11	0.53	1.05	1.07	0.39	0.78	0.48	0.51	0.71	0.62
S (LSD 0.05%)		10.11	0.53	1.05	1.07	0.39	0.78	0.48	0.51	0.71	0.62
α-Toc (LSD 0.05%)		14.3	0.75	1.49	1.51	0.56	1.10	0.67	0.73	1.00	0.88

*, **, and ***Significant at 0.05, 0.01 and 0.001 levels, respectively; ns, non-significant; V, Varieties; S, Salinity; α-toc, Alpha tocopherol.

The saline regimen markedly enhanced the concentration of leaf Na^+^ ions. However, the trend of Na^+^ ion accumulation in the leaves of both okra varieties (Noori and Sabzpari) differed non-significantly. Moreover, the response of both okra varieties was also non-significant for the foliar spray of α-tocopherol in decreasing Na^+^ ion concentration (Table 3 and Figure 7C).

**FIGURE 8:**
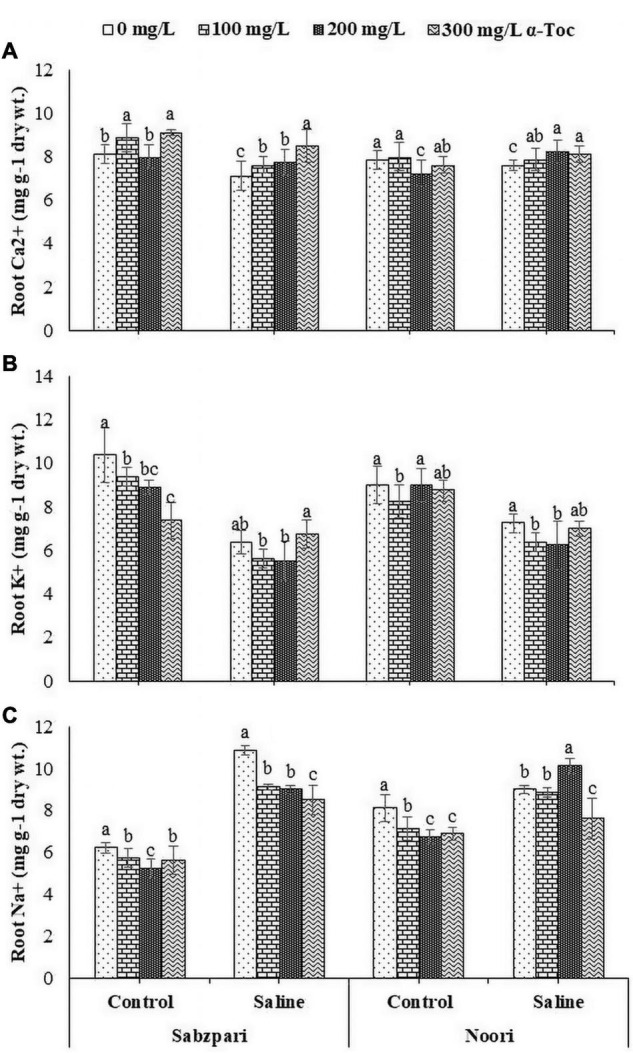
Root Ca^2+^ **(A)**, Root K^+^ **(B)** Root Na^+^ **(C)** of okra varieties sprayed with different levels of α-tocopherol under saline conditions. Values represent means ± S.D. Significant differences among row spacing were measured by the least significant difference (LSD) at *p* > 0.05 and indicated by different letters.

Both okra varieties showed non-significant differences in terms of root ion content, i.e., Ca^2+^, K^+^, and Na^+^. The addition of salt stress non-significantly reduced the root Ca^2+^ content in Sabzpari, while an increase was observed in Noori. Salt stress markedly decreased the K^+^ ion content in the roots of both tested varieties of okra. Root medium salt stress significantly enhanced the root Na^+^ ion concentration in both tested varieties. Sprays of α-tocopherol did not significantly affect the concentrations of root Ca^2+^ and K^+^. Foliar application of α-tocopherol at 300 mg L^–1^ significantly reduced the inhibitory effect of Na^+^ ion concentration in both okra varieties under saline stress (Table 3 and Figures 8A–C).

### Soluble Sugars and Protein Content

**FIGURE 9:**
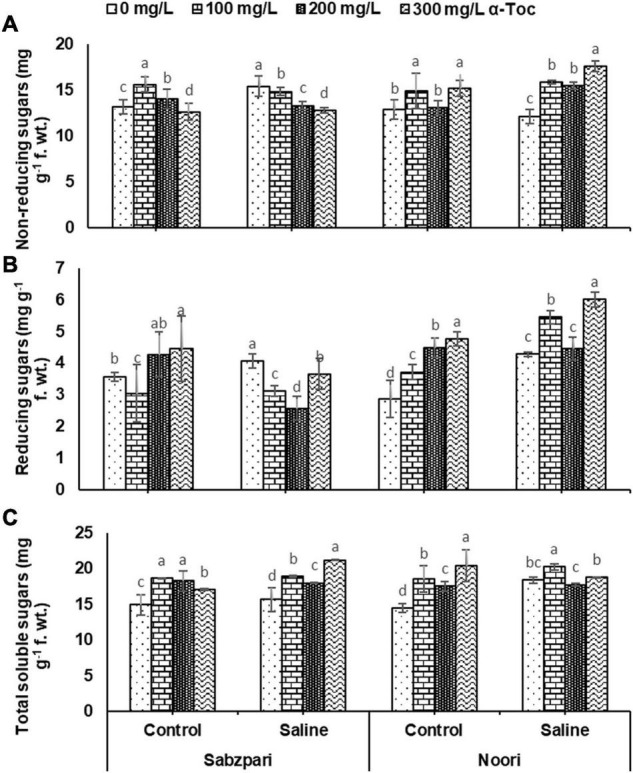
Non-reducing sugars **(A)**, Reducing sugars **(B)**, Total soluble sugars **(C)** of okra varieties sprayed with different levels of α-tocopherol under saline conditions. Values represent means ± S.D. Significant differences among row spacing were measured by the least significant difference (LSD) at *p* > 0.05 and indicated by different letters.

Salt stress increased the concentration of total soluble sugars and reducing and non-reducing sugars in both varieties of okra. Both varieties (Noori and Sabzpari) differed non-significantly in terms of total soluble sugars and non-reducing sugars. However, reducing sugar content showed a significant difference in the performance of both okra varieties, as Noori was superior in the accumulation of reducing sugars than Sabzpari under salt and non-salt conditions. A prominent increase was observed in the concentration of total soluble sugars and non-reducing sugars in both varieties of okra due to α-tocopherol spray. However, the effect of exogenously applied α-tocopherol as foliar spray on okra plants was non-significant in enhancing the reducing sugar content (Table 3 and Figures 9A–C).

**FIGURE 10:**
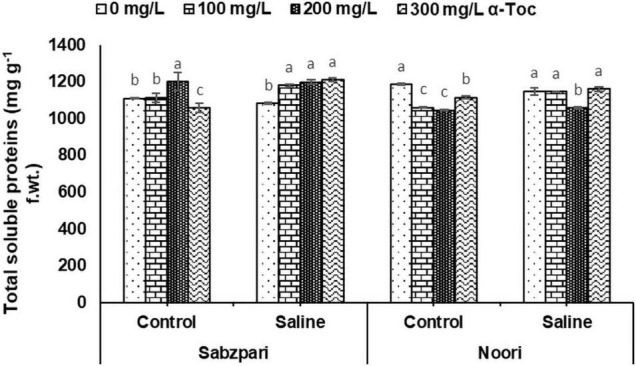
Total soluble proteins of okra varieties sprayed with different levels of α-tocopherol under saline conditions. Values represent means ± S.D. Significant differences among row spacing were measured by the least significant difference (LSD) at *p* > 0.05 and indicated by different letters.

Both okra varieties showed significant differences in terms of total soluble protein content. Noori was superior to Sabzpari in this attribute. Total soluble protein content was markedly enhanced under saline stress. The effect of foliar-applied α-tocopherol on enhancing the total soluble protein content was not remarkable, whereas a significant interaction was observed among the varieties, NaCl treatment and foliar spray of α-tocopherol (Table 3 and Figure 10).

## Discussion

Plant growth is an important criterion to evaluate salinity tolerance in plants, as higher accumulation of NaCl in plant tissues adversely affects plant growth (Kachout et al., 2016). In the current study, suppression of growth in terms of root and shoot fresh and dry weights and their lengths was observed for okra plants under salinity stress. Reduced growth is directly linked with reduced photosynthetic activity and stunted growth of vegetative plant parts under saline regimes. Decreased growth is linked to blocked activity of photosynthesis and inhibited uptake of essential ions (Farooq et al., 2017). However, foliar fertigation of α-tocopherol alleviated the adverse effects of salinity by improving these growth attributes. This shows that α-tocopherol stimulates various signaling factors involved in growth stimulation. Improved photosynthesis is also linked with upregulation of α-tocopherol, which is linked with enhanced growth (Orabi and Abdelhamid, 2016). Abiotic factors, including salinity, reduce the photosynthetic rate. Elevated levels of NaCl also hamper stomatal function, uptake of CO_2_ and ultimately photosynthesis (Hnilickova et al., 2021). In the present study, a reduction in photosynthetic pigments was markedly observed under salinity stress in the tested okra varieties. Salinization disturbs the biosynthesis of photosynthetic pigments by disorganizing the structure of chlorophyll (Kumar et al., 2013). The process of photosynthesis comprises various components, and damage by salinity stress at any stage may reduce the plant’s overall photosynthetic rate. However, different levels of α-tocopherol applied as foliar spray significantly altered the content of photosynthetic pigments, including chlorophyll *a* and *b*, β-carotenes, total carotenoids and total chlorophyll of okra, under salinity stress. α-Tocopherol protects chloroplast structure from salinity-induced photoinhibition (Sh, 2014), as the effects of salinity on photosynthesis may involve inhibition of electron transport and inactivation of photosystem II (PSII) reaction centers (Mehta et al., 2010). α-Tocopherol also enhances the production of chlorophyll pigments by acting as an antioxidant and combating salinity-induced oxidative bursts as α-tocopherol is synthesized in plastids (Surówka et al., 2020).

Photosynthetic absorption of solar energy and CO_2_ fixation are directly linked with fluctuations in CO_2_ assimilation (Joshi et al., 2018). The rate of photosynthesis can be affected by environmental factors at all levels, and many researchers have reported a reduction in the rate of photosynthesis due to salinity. Stomatal regulation is extremely sensitive to salinity stress, which results in a reduced photosynthetic rate, transpiration rate, chlorophyll content and stomatal conductivity (Tang et al., 2020). In the present study, salinity stress markedly reduced gas exchange characteristics (*A, E, A/E, gs, C_*i*_* and *C_*i*_/C_*a*_*) in the tested okra varieties. Reduced stomatal conductance is linked with reduced intercellular CO_2_, which decreases the activity of several enzymes, including RUBISCO. However, gas exchange characteristics (*A, E, A/E, gs, C_*i*_*, and *C_*i*_/C_*a*_*) were improved due to foliar spray of α-tocopherol under salinity stress in okra plants in the current study. This augmentation in the conductance efficiency of okra plants by exogenous supply of α-tocopherol is due to its antioxidative property. As an antioxidant, it protects photosystems and plastid membranes from lipid peroxidation by scavenging salinity-induced oxidative bursts (Fritsche et al., 2017). All these findings recommend the salinity mitigation role of α-tocopherol as a shielding agent by protecting chlorophyll pigments from photoinhibition. Agricultural crops suffer severe nutritional disorders due to competition among Na^+^, Ca^2+^, and K^+^ ions (Singh et al., 2021). In this study, higher concentrations of Na^+^ were observed in the leaf and root tissues of okra plants under saline regimes. Enhanced Na^+^ concentrations disturb photosynthesis, plant metabolism, and enzymatic activities and thus affect overall crop productivity (Ashraf and Harris, 2013). Reduced uptake of Ca^2+^ is correlated with an enhanced concentration of Na^+^. Foliar spray of α-tocopherol proved effective in minimizing the toxic concentrations of Na^+^ ions in root and leaf tissues of okra plants, suggesting the role of α-tocopherol in osmotolerance by lowering Na^+^ levels in plants (Sadak and Dawood, 2014). Foliar spray of alpha tocopherol enhanced the concentration of K^+^ and Ca^2+^ ion contents in root and leaf of okra plants in the present study although, root medium applied salt stress decreased the concentration of these ions in root and leaf tissues of okra plants. High salt (NaCl) concentrations in soil and groundwater affect the ability of plants to take up K^+^ and adversely affect enzymatic activities, cellular turgor and protein biosynthesis (Ahmed et al., 2021). Calcium plays a vital role in plants by providing structural stability and as a signaling molecule, but high concentrations of toxic salts reduce the uptake of Ca^2+^. α-Tocopherol supplementation helps lower Na^+^ ion content and enhances the uptake of K^+^ under abiotic stress and non-stressed conditions (Ahmed et al., 2021). Total soluble proteins function as compatible solutes and are upregulated during abiotic stress conditions (Singh and Husen, 2019). In the current study, salt stress conditions enhanced the total soluble proteins in okra. On the other hand, foliar application of α-tocopherol induced a non-significant rise in soluble protein content. Alpha tocopherol is involved in the biosynthesis of proteins. A positive correlation also exists between exogenous application of α-tocopherol and protein biosynthesis (Sadak and Abdelhamid, 2015). It is hypothesized that enhanced levels of total soluble proteins enhance SOD activities in plants and minimize adverse effects of ROS generated under salinity stress.

The accumulation of carbohydrates (such as sugars) under stress conditions is one of most important plant response mechanisms to attain stress tolerance by osmoprotection, osmoregulation, carbon storage, and working against ROS. Total soluble sugars and reducing sugars were remarkably enhanced in the tested okra varieties under salinity. A significant increase was observed in total soluble sugars and non-reducing sugars due to α-tocopherol spray. Salinity tolerance may have been achieved due to the accumulation of carbohydrates as a protection mechanism (Sadiq et al., 2017). The accumulation of sugars due to abiotic stresses is thought to be a symbol of membrane damage due to stress-induced ROS (Islam et al., 2016). Alpha tocopherol helps quench ROS and is also responsible for the higher buildup of carbohydrates in plants (Sadak et al., 2015) under salinity stress conditions, the accumulation of sugars helps plants adjust osmotically. Plants have been attributed to adaptation by an increase in carbohydrate levels in response to stresses. In addition to osmoregulators, soluble sugars may act as osmoprotectants for proteins under stressed conditions.

## Conclusion

Foliar application of α-tocopherol proved effective in alleviating salinity-induced damage in okra by enhancing growth, photosynthetic pigments, and leaf gas exchange attributes, possibly by protecting chloroplasts due to its antioxidant potential. Among the tested okra varieties, Noori showed enhanced tolerance against salinity, and 300 mg L^–1^ α-tocopherol was more effective. Thus, this study points to the use of the okra variety Noori to be grown in saline soils with foliar spray of α-tocopherol (300 mg L^–1^) to increase okra production under field conditions. Exogenous application of α-tocopherol is also recommended to apply on other crops to alleviate salinity induced damages. Other modes of exogenous application of α-tocopherol e.g., seed priming can also be tested to cope with salinity stress in other crops.

## Data Availability Statement

The original contributions presented in the study are included in the article/supplementary material, further inquiries can be directed to the corresponding author/s.

## Author Contributions

MN and MS conceived the idea. XW and AM provided the technical expertise to strengthen the basic idea. SF and SB helped in the collection of data and its analysis. AM helped in the proofreading and discussion of the manuscript. All authors have read the first draft, helped in revision and approved the article.

## Conflict of Interest

The authors declare that the research was conducted in the absence of any commercial or financial relationships that could be construed as a potential conflict of interest.

## Publisher’s Note

All claims expressed in this article are solely those of the authors and do not necessarily represent those of their affiliated organizations, or those of the publisher, the editors and the reviewers. Any product that may be evaluated in this article, or claim that may be made by its manufacturer, is not guaranteed or endorsed by the publisher.
